# Differential effects of eplerenone versus amlodipine on muscle metaboreflex function in hypertensive humans

**DOI:** 10.1111/jch.14333

**Published:** 2021-08-25

**Authors:** Poghni A. Peri‐Okonny, Alejandro Velasco, Hamza Lodhi, Zhongyun Wang, Debbie Arbique, Beverley Adams‐Huet, Gary Iwamoto, Jere H. Mitchell, Masaki Mizuno, Scott Smith, Wanpen Vongpatanasin

**Affiliations:** ^1^ Hypertension Section University of Texas Southwestern Medical Center Dallas Texas USA; ^2^ Department of Clinical Sciences University of Texas Southwestern Medical Center Dallas Texas USA; ^3^ Department of Cell Biology University of Texas Southwestern Medical Center Dallas Texas USA; ^4^ Cardiology Division University of Texas Southwestern Medical Center Dallas Texas USA; ^5^ Department of Health Care Sciences University of Texas Southwestern Medical Center Dallas Texas USA

**Keywords:** exercise, hypertension, metaboreflex, sympathetic nervous system

## Abstract

Numerous studies have demonstrated that sympathetic nervous system overactivation during exercise in hypertensive rodents and humans is due, in part, to an exaggerated reflex response known as the exercise pressor reflex. Our prior studies have implicated a key role of mineralocorticoid receptor activation in mediating an augmented exercise pressor reflex in spontaneously hypertensive rats, which is mitigated by blockade with eplerenone. However, the effect of eplerenone on exercise pressor reflex has not been assessed in human hypertension. Accordingly, the authors performed a randomized crossover study to compare the effects of eplerenone to another antihypertensive drug from a different class amlodipine on sympathetic nerve activity (SNA) in 14 patients with uncomplicated hypertension. The authors found that amlodipine unexpectedly augmented the increase in SNA during the second minute of isometric handgrip, which persisted into the post‐exercise circulatory arrest period (∆ SNA, from rest of 15 ± 2 vs. 9 ± 2 vs. 10 ± 2 bursts/min, amlodipine vs. baseline vs. eplerenone, respectively, *p* < .01), suggesting an exaggerated muscle metaboreflex function. Eplerenone did not alter sympathetic responses to exercise or post‐exercise circulatory arrest in the same hypertensive individuals. In conclusions, our studies provide the first direct evidence for a potentially unfavorable potentiation of muscle metaboreflex by amlodipine during isometric handgrip exercise in hypertensive patients whereas eplerenone has no significant effect. Our study may have clinical implications in terms of selection of antihypertensive agents that have the least detrimental effects on sympathetic neural responses to isometric exercise.

## 1 INTRODUCTION

A large body of evidence in rodents and humans have indicated that hypertension is associated with overactivation of the sympathetic nervous system during exercise, which is independent of resting sympathetic nerve activity (SNA).^1^, ^2^, ^3^, ^4^ Normally, exercise produces intensity‐dependent increases in heart rate (HR), cardiac output (CO), and blood pressure (BP), mediated at least in part by increases in SNA. A reflex arising in the contracting skeletal muscle, known as exercise pressor reflex (EPR), is known to play a major role in driving SNA during exercise. The EPR can be activated either by *metaboreceptors*, which are stimulated slowly by the metabolic byproducts of muscle contraction (ie, metaboreflex), or *mechanoreceptors*, which respond quickly to mechanical deformation of the muscle fibers (ie, mechanoreflex).^5^

Studies in young spontaneously hypertensive rat (SHR), a standard rat model of primary hypertension before development of heart failure, demonstrated that both the metabo‐ and mechanoreflex is overactive.^4^, ^6^ Studies in hypertensive patients have also suggested augmented EPR,^7^ particularly in muscle metaboreflex function.^8^, ^9^ While the precise mechanisms underlying this observation are unknown, an increasing number of studies have demonstrated a potentially important role for central mineralocorticoid receptors (MR) in mediating sympathetic nervous system overactivation even in the presence of normal circulating aldosterone levels.^10^, ^11^ Furthermore, our recent studies have implicated a key role for MR activation in mediating the augmented EPR observed in hypertensive rats^12^ which was reversed by treatment with the MR antagonist eplerenone.^10^ However, the role of MR in modulating EPR function in human hypertension has not been assessed.

Therefore, we conducted a randomized crossover study to assess the effects of eplerenone, a selective MR antagonist, on SNA and BP during static and dynamic exercise in patients with uncomplicated hypertension. To further determine if the potential effects of eplerenone are specific to its drug class, we likewise compared the effects of eplerenone to the commonly used antihypertensive drug amlodipine, a calcium channel blocker known to have neutral effects on resting SNA.^13^

## 2 METHODS

All experimental protocols were approved by the Institutional Review Board of the University of Texas Southwestern Medical Center. Written, informed consent was obtained from all of the patients.

### 2.1 Patients

Fourteen patients with essential hypertension participated in the study. Eligible patients included untreated hypertensive patients with BP between 140–159/90–99 mm Hg on three determinations by oscillometric technique (CE0050, Welch Allyn, Skaneateles Falls, NY, USA) in the seated position. Treated hypertensive patients with BP between 130–159/90–99 mm Hg were also included in the study after all antihypertensive medications were withdrawn for at least 1 week before the study. Patients had no history of heart disease, diabetes mellitus, or evidence of target organ damage such as left ventricular hypertrophy by electrocardiography or chronic kidney disease. Five patients had never been treated with antihypertensive agents before the study due to lack of insurance. Five patients were treated with Lisinopril, two of which were given in combination of hydrochlorothiazide and two in combination with amlodipine. One patient treated with amlodipine alone, one with metoprolol alone, one with enalapril alone, and one with losartan alone. None of patients were treated with MR antagonists before the study.

### 2.2 Measurements

#### 2.2.1 Measurement of sympathetic nerve activity by microneurography

Multiunit recordings of SNA were obtained with unipolar tungsten microelectrodes inserted into muscle fascicles of the peroneal nerve by microneurography.^12^ Neural signals were amplified, filtered (bandwidth 700–2000 Hz), rectified, and integrated to obtain mean voltage neurograms. Recordings were considered acceptable based on well‐defined criteria that discriminate muscle SNA from other neural signals including skin SNA and muscle spindle activity.^13^ Muscle SNA was expressed as burst frequency (bursts/min) and total activity (burst frequency × mean burst amplitude).

#### 2.2.2 Cardiac output

Cardiac output was measured at rest and during handgrip exercise by thoracic electrical bioimpedance (BioZ, CardioDynamics, San Diego, CA, USA) as previously described.^14^ Stroke volume (SV) was derived from change in impedance/time measured during electrical systole. Cardiac output was determined as the product of SV and HR.

### 2.3 Exercise protocols

#### 2.3.1 Static handgrip exercise (SHG)

To assess SNA response to isometric exercise, SHG were performed at 30% maximal voluntary contraction (MVC) for 2 min while SNA and BP is monitored as above.^15^ During the last 5 s of static HG exercise, a pneumatic cuff on the upper exercising arm were inflated to 50 mm Hg above systolic BP for 2 min (Post‐Handgrip Exercise Circulatory Arrest, PECA) to isolate the muscle metaboreflex. MVC for each patient was designated as the greatest of at least three maximal squeezes of a handgrip dynamometer (Stoelting, Chicago, IL, USA).

#### 2.3.2 Arm cycling

To assess SNA response to dynamic exercise, patients were asked to perform dynamic arm cycling. The patient's dominant hand was strapped to one of the pedals of the arm ergometer (Exerpeutic 7101 Active Cycle Mini Exercise Bike, PARADIGM Health & Wellness, City of Industry, CA, USA). Then, the patients were asked to move the arm ergometer at 40 replications per minute (r.p.m.) for 3 min (70 watts) followed by 80 r.p.m. for 3 min (140 watts) without resting period between two frequencies.

Ratings of perceived exertion (RPE) were obtained at the end of each exercise by using a 6‐ to 20‐unit Borg scale. Each exercise intervention was separated by at least 15 min to allow BP to return to baseline. At the end of exercise interventions, a cold pressor test (CPT) was performed in all patients by immersing the dominant hand in an ice water bath (4°C) up to the wrist for 2 min to determine if the potential effects of eplerenone and amlodipine on SNA during handgrip exercise are limited to physical stress or all stressful stimuli.

### 2.4 Test protocols

All patients underwent measurement of BP, HR, CO, SV, total peripheral resistance (TPR, defined as Mean arterial pressure [MAP]/CO), and SNA in supine position at rest and in response to arm cycling, static handgrip, PECA, and CPT at baseline. Then, they were randomized to receive 8 weeks of eplerenone (50–200 mg/day) or amlodipine (2.5–10 mg/day), using a single‐blind crossover design without washout between treatment. This dose range was chosen based on a previous study showing similar reduction in office BP in older patients with isolated systolic hypertension.^16^ Each patient was followed every 2 weeks for measurement of office BP and serum potassium (K). The doses of eplerenone and amlodipine were titrated to achieve office BP of < 140/90 mm Hg in the same patient. Muscle SNA was measured after 8 weeks of eplerenone and after 8 weeks of amlodipine. Then, patients were given drug treatment in the remaining arm for another 8 weeks. Body weight, serum K, serum sodium (Na), serum creatinine (Cr), fasting plasma glucose, total cholesterol, HDL‐cholesterol, and triglyceride levels were measured at baseline, after 8 weeks of eplerenone, and after 8 weeks of amlodipine. Analysis of these variables was performed without the knowledge of treatment each patient had received.

## 3 STATISTICAL ANALYSIS

Mixed‐effects linear models were used to conduct the repeated measures analysis to assess differences between the baseline period, amlodipine, and eplerenone phases. Time points measured during static hand grip, arm cycling, or CPTs were included in the mixed‐models as an added repeated factor. The model covariance structure was evaluated and selected based on Akaike Information Criterion. Contrasts from these models were used for pair‐wise comparisons. Treatment order was also assessed in the models and no effect of treatment order on any outcome variables was found (all interaction *p* values are > .1). Because percentage of change in total activity of SNA (product of average bursts/min and mean burst amplitude) was skewed, the data were analyzed after a natural logarithmic transformation. The 0.05 level of significance was used for model main effects and 0.1 for assessing interactions; pair‐wise tests are adjusted for multiple testing at the level of 0.01. Data in the tables are summarized as mean and standard deviation and results reported in the text and figures are expressed as mean and SEM. Statistical analysis was performed with SAS version 9.4 (SAS Institute Inc., Cary, NC, USA).

## 4 RESULTS

Baseline characteristics and biochemical changes during treatment with amlodipine and eplerenone are shown in Table 1. The average daily dose of amlodipine and eplerenone used in the study was 5.5±0.7 mg and 119.6±16 mg, respectively.

**TABLE 1:** Baseline characteristics of participants (*n* = 14) and effects of amlodipine versus eplerenone on metabolic parameters

Variables	Baseline	Amlodipine	Eplerenone	Mixed model *p* value^a^
Age	51.1 ± 8.5			
Female (%)	21%			
Ethnicity	5 African Americans, 8 Caucasians, 1 Hispanic			
Body mass index (kg/m^2^)	31.8 ± 5.8	32.1 ± 5.9	31.1 ± 5.2	.43
Body weight (kg)	96.8 ± 20.1	97.6 ± 20.3	94.1 ± 16.9	.42
Serum Na (mmol/L)	140.2 ± 3.7	141.3 ± 6.2	139.7 ± 3.1	.46
Serum K (mmol/L)	4.2 ± 0.3	4.1 ± 0.4	4.3 ± 0.3^*^	.05
Serum Cr (mg/dl)	0.98 ± 0.19	0.96 ± 0.17	0.97 ± 0.20	.60
Fasting plasma glucose (mg/dl)	105.0 ± 13.1	104.9 ± 11.3	102.8 ± 9.8	.70
Total cholesterol (mg/dl)	177.4 ± 23.2	187.2 ± 29.1	193.6 ± 37.5	.11
Triglycerides (mg/dl)	139.2 ± 42.8	143.6 ± 58.6	143.2 ± 48.9	.91
HDL‐Cholesterol (mg/dl)	45.1 ± 10.4	44.3 ± 10.7	44.4 ± 9.4	.87
Seated systolic BP (mm Hg)	136.3 ± 10.8	132.1 ± 8.3	131.8 ± 13.8	.19
Seated diastolic BP (mm Hg)	89.6 ± 6.4	85.6 ± 4.1	87.8 ± 5.7	.06
Seated heart rate (beats/min)	79.8 ± 14.6	77.5 ± 11.9	81.8 ± 14.3	.40

Data are mean value ± standard deviation.

^*^ *p* = .01 versus amlodipine.

^a^ For comparison between baseline and two treatment phases.

Serum potassium (K) was significantly increased after eplerenone treatment when compared to amlodipine. Diastolic BP tended to be lower after eplerenone and amlodipine but the difference did not reach statistical significance (Table 1). There was no change in body weight, body mass index, serum Cr, serum Na, fasting plasma glucose, total cholesterol, HDL‐cholesterol, or triglyceride levels during either drug treatment.

**FIGURE 1:**
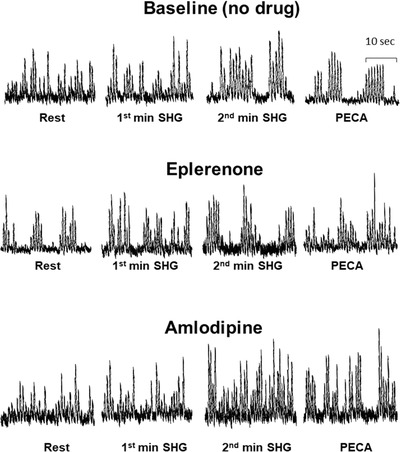
Representative SNA (sympathetic nerve activity) from one hypertensive patient at baseline (no drug), after 8 weeks of eplerenone (middle panel), and after 8 weeks of amlodipine (bottom panel)

**FIGURE 2:**
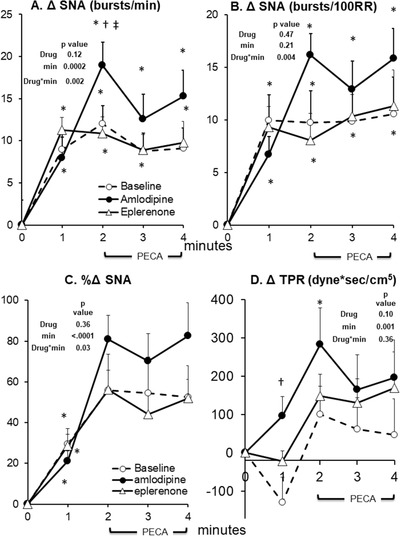
SNA (sympathetic nerve activity) and total peripheral resistance (TPR) response to static handgrip at baseline (no drug, *n* = 14), after 8 weeks of amlodipine (*n* = 14) and after 8 weeks of eplerenone (*n* = 14). Data are Mean ± SD. *p*‐values of linear mixed model are shown. ^*^ *p* < .01 versus rest (minute 0), ^†^ *p* < .01 versus Baseline (no drug), ^‡^ *p* < .01 versus eplerenone

**FIGURE 3:**
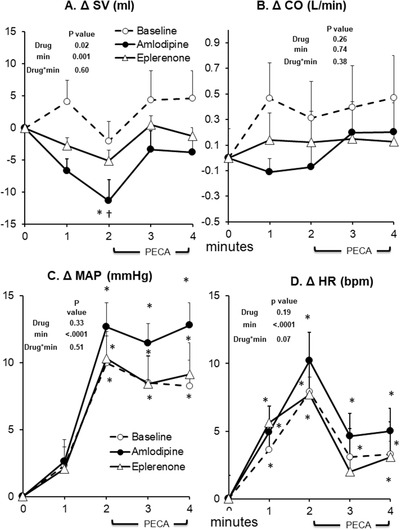
Cardiovascular and blood pressure responses to static handgrip at baseline (no drug, *n* = 14), after 8 weeks of amlodipine (*n* = 14), and 8 weeks of eplerenone (*n* = 14). Data are Mean ± SD. *p*‐values of linear mixed model are shown. ^*^ *p* < .01 versus rest (minute 0), ^†^ *p* < .01 versus Baseline (no drug), ^‡^ *p* < .01 versus eplerenone

Microneurographic recording of SNA was unsuccessful in one participant during eplerenone phase; however, all other available data for this participant was included in the analyses. Both eplerenone and amlodipine had no effect on resting SNA, SV, CO, TPR, or MAP (Table 2). Eplerenone induced a small but significant increase in resting HR while amlodipine had no significant effect (Table 2). At baseline without antihypertensive drug treatment, static handgrip induced a significant increase in SNA, HR, and MAP in hypertensive patients (Figures 1, 2, 3). As expected, when circulation to the exercising arm was prevented by cuff inflation above systolic BP at handgrip end, HR fell towards resting values during minute 3 and 4 while muscle SNA, and MAP remained elevated due to muscle metaboreflex activation.^15^ There were no significant changes in TPR or SV during static handgrip or PECA. Cardiac output tended to increase during the first minute of handgrip (minute 1) and PECA (minute 4, *p* < .05 vs. minute 0, Figure 3B), but the overall mixed model *p* value is not significant. Eplerenone had no effect on SNA, SV, CO, HR, TPR, or MAP during static handgrip and PECA. In contrast, amlodipine augmented the increase in SNA, which was evident beginning from the second minute of handgrip (ΔSNA from rest of 19 ± 2 vs. 12 ± 2 vs. 11 ± 2 bursts/min, amlodipine vs. baseline vs. eplerenone, respectively, both *p* < .01) persistent into the PECA phase in minutes 3–4 (ΔSNA from rest of 15 ± 2 vs. 9 ± 2 vs. 10 ± 2 bursts/min, amlodipine vs. baseline vs. eplerenone, respectively, pairwise *p* < .05, Figures 1 and 2). The augmented increase in SNA induced by amlodipine was accompanied by an exaggerated rise in HR during the same period. Furthermore, amlodipine induced a significant decrease in SV (mixed model *p* value of .02) during static handgrip in the same hypertensive patients (Figure 3A). The increase in TPR during static handgrip tended to be augmented with amlodipine (pairwise *p* value < .05 vs. no drug during the first minute (Figure 2D), though the overall mixed model p value was not significant. Amlodipine had no significant effects on CO or MAP during static handgrip or PECA (Figure 3B–C).

**TABLE 2:** Resting hemodynamic and sympathetic nerve responses to amlodipine and eplerenone

Variables	Baseline (No drug)	Amlodipine	Eplerenone	Mixed model *p* value^a^
Supine MAP, mm Hg	108.8 ± 7.3	105.7 ± 7.4	108.2 ± 10.7	.41
Supine HR, bpm	67.9 ± 7.0	70.1 ± 7.8	71.6 ± 8.1	.13
SNA, bursts/min	41.5 ± 10.4	41.0 ± 11.7	43.8 ± 13.2	.84
SNA, bursts/100RR	61.2 ± 14.8	58.0 ± 16.2	58.7 ± 16.8	.81
Stroke Volume (ml)	70.7 ± 22.5	80.1 ± 31.5	65.4 ± 13.8	.25
Cardiac output (L/min)	4.93 ± 1.34	5.50 ± 1.89	4.64 ± 0.82	.19
Total Peripheral Resistance (dyne*s/cm^5^)	1872 ± 567	1,646 ± 388	1,925 ± 417	.10
Maximal force (kg)	40.3 ± 16.1	37.4 ± 10.2	39.0 ± 12.7	.73
Borg scale				
‐ During static handgrip	14.6 ± 2.3	15.5 ± 1.7	16.1 ± 1.7	.06
‐ During active arm cycling	11.9 ± 2.5	11.3 ± 2.4	12.1 ± 2.8	.48

Data are mean value ± standard deviation.

*Abbreviations*: MAP, mean arterial pressure; HR, heart rate; SNA, sympathetic nerve activity.

^a^ For comparison between baseline and two treatment phases.

**FIGURE 4:**
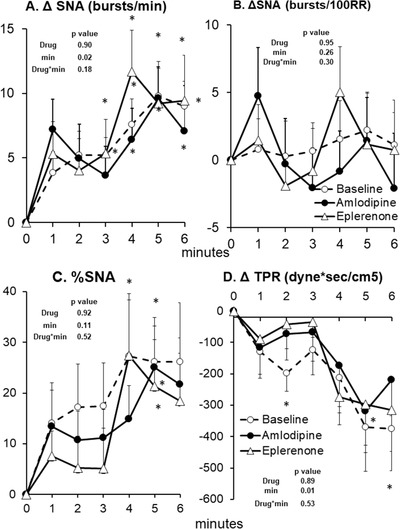
SNA and total peripheral resistance (TPR) responses to arm cycling exercise at baseline (no drug, *n* = 14), after 8 weeks of amlodipine (*n* = 14) and after 8 weeks of eplerenone (*n* = 14). Data are Mean ± SD. *p*‐values of linear mixed model are shown. ^*^ *p* < .01 versus rest (minute 0), ^†^ *p* < .01 versus Baseline (no drug), ^‡^ *p* < .01 versus eplerenone

**FIGURE 5:**
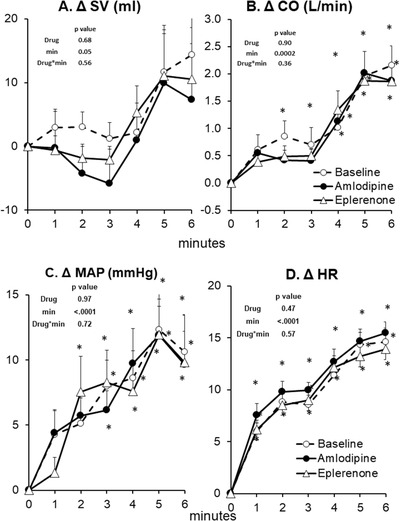
Cardiovascular and blood pressure responses to dynamic arm cycling at baseline (no drug, *n* = 14), after 8 weeks of amlodipine (*n* = 14) and after 8 weeks of eplerenone (*n* = 14). Data are Mean ± SD. *p*‐values of linear mixed model are shown. ^*^ *p* < .01 versus rest (minute 0), ^†^ *p* < .01 versus Baseline (no drug), ^‡^ *p* < .01 versus eplerenone

**FIGURE 6:**
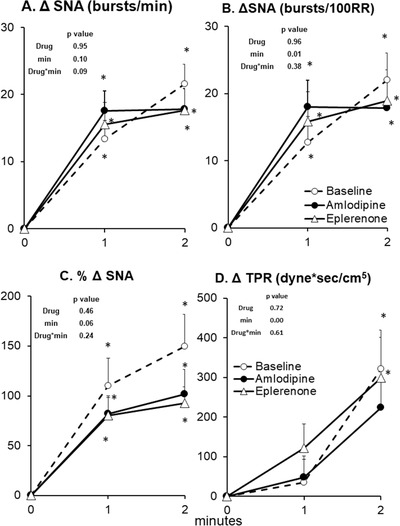
SNA and TPR responses to cold pressor test at baseline (no drug, *n* = 14), after 8 weeks of amlodipine (*n* = 14) and after 8 weeks of eplerenone (*n* = 14). Data are Mean ± SD. *p*‐values of linear mixed model are shown. ^*^ *p* < 0.01 versus rest (minute 0), ^†^ *p* < 0.01 versus Baseline (no drug), ^‡^ *p* < 0.01 versus eplerenone

**FIGURE 7:**
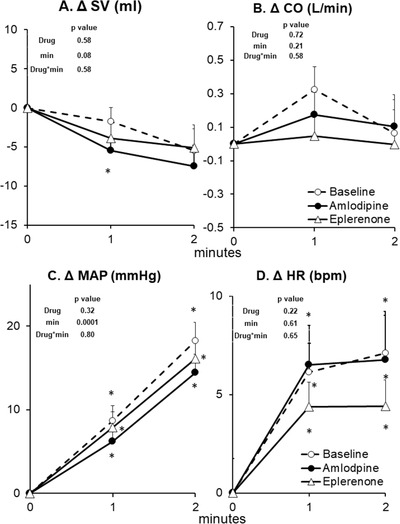
Cardiovascular and BP responses to cold pressor test at baseline (no drug, *n* = 14), after 8 weeks of amlodipine (*n* = 14) and after 8 weeks of eplerenone (*n* = 14). Data are Mean ± SD. *p*‐values of linear mixed model are shown. * *p* < .01 versus rest (minute 0), ^†^ *p* < .01 versus Baseline (no drug), ^‡^ *p* < 0.01 versus eplerenone

Arm cycling induced a progressive rise in SNA, MAP, HR, and CO as well as a progressive decline in TPR (Figures 4 and 5). Both eplerenone and amlodipine had no effect on sympathetic and hemodynamic responses to arm cycling. The CPT induced a progressive rise in SNA, MAP, HR, and TPR while SV tended to be reduced (Figures 6 and 7). Both eplerenone and amlodipine had no effect on sympathetic and hemodynamic responses to CPT. Both eplerenone and amlodipine also had no significant effects on maximal handgrip force or RPE (Borg scale) during both static handgrip and active arm cycling (Table 2).

## 5 DISCUSSION

The major findings of the study are two‐fold. First, the selective MR antagonist eplerenone had no significant effects on SNA and BP responses to static and dynamic arm exercise in hypertensive patients. Second, a commonly used calcium channel blocker, amlodipine, unexpectedly potentiated the rise in SNA and HR during static handgrip and post‐handgrip circulatory arrest (a metaboreflex isolating experimental procedure). This effect of amlodipine was not observed during dynamic arm cycling exercise, suggesting a selective metaboreflex sensitization during static exercise.

The mechanisms by which amlodipine induced sympathetic activation during handgrip exercise are unknown. Amlodipine is a long‐acting dihydropyridine calcium channel blocker considered to be the one of the first‐line drug treatments for hypertension.^17^ We chose amlodipine as a control due to prior report of neutral effects on resting SNA in humans.^18^ In our study, resting SNA was also unaffected by amlodipine and the augmented rise in SNA and HR was observed only after the first minute of static handgrip. This slow time course is consistent with metaboreflex activation as group IV muscle afferents, which are typically activated by muscle acidosis and metabolic byproducts of muscle contraction, display long response latencies in increasing sympathetic outflow and BP.^19^, ^20^, ^21^ Although an amlodipine‐induced augmentation of SNA and HR responses during handgrip may be due to impaired baroreflex function, this is unlikely as similar responses were not observed during arm cycling exercise or the CPT. Furthermore, previous studies have not shown any effects of amlodipine on baroreflex control of SNA and HR in humans.^13^ Since amlodipine did not potentiate the increase in SNA or HR during dynamic arm cycling or the CPT, this suggests selective metaboreflex sensitization by amlodipine. Potentiation of central command, a central neural drive associated with the volitional component of exercise, is another possible mechanism by which amlodipine may have augmented the rise in SNA and HR during static exercise. However, this is unlikely since the elevation in HR induced by amlodipine was observed during PECA when the patients were no longer performing handgrip exercise. In addition, RPE by the Borg scale were not altered by amlodipine; an indirect indication that central command input was not different between experimental trials.

Previous studies in normal humans and dogs have demonstrated that elevated BP during muscle metaboreflex activation is related to elevations in CO rather than increases in total peripheral resistance.^22^, ^23^ In the presence of hypertension, however, metaboreflex‐induced increases in CO and SV are markedly attenuated and elevated TPR plays a larger role in maintaining BP during exercise.^24^ Our study extended previous observations by demonstrating that amlodipine further exaggerated the rise in TPR during isometric exercise, which may be deleterious if sustained over a prolonged period in hypertensive individuals.

The mechanisms underlying the amlodipine‐induced sensitization of the metaboreflex during static exercise remain unknown. Amlodipine may induce greater increases in muscle lactate or acidosis, which are major stimulators of group IV muscle afferents during static handgrip. However, this is unlikely as previous studies did not show an augmented increase in lactate production in isolated guinea pig hearts subjected to coronary ischemia in the presence of amlodipine.^25^ Similarly, Gillies and coworkers demonstrated no significant impact of amlodipine on plasma lactate levels during leg cycling exercise in hypertensive patients.^26^ Interestingly, in the same study, amlodipine was found to reduce resting BP without lowering BP during isometric exercise which is similar to the findings of our present study.^26^

Amlodipine is an antagonist of the voltage‐dependent L‐type calcium channel (LTCC). At least four isoforms of LTCC have been identified (Ca_v_1.1, Ca_v_1.2, Ca_v_1.3, and Ca_v_1.4). Ca_v_1.3 is expressed together with Ca_v_1.2 in many tissues, including dorsal root ganglion (DRG) and spinal neurons.^27^, ^28^ Dysregulation of Ca_v_1.2 and Ca_v_1.3 have been implicated in the pathogenesis of neuropathic pain.^27^, ^28^, ^29^ Since muscle afferent neurons which are residing in the DRG serve a very important role in the afferent arm of sympathetic reflexes evoked by muscle contraction,^19^, ^21^ future studies are needed to determine if augmented muscle metaboreflex responses to static exercise induced by amlodipine is related to preferential inhibition of particular LTCC isoforms in the DRG of hypertensive patients or rodents.

Our study demonstrated no significant effects of eplerenone in modulating sympathetic neural and pressor response to both isometric and dynamic exercise in hypertensive patients which is in contrast with the sympatho‐inhibitory effect of this drug class demonstrated in our previous rodent studies.^10^ There are several potential explanations underlying this discrepancy. First, the dose of eplerenone used in our study may be too low as it had no significant impact on resting BP though its effect on serum potassium was detected, suggesting its action on the distal nephron. Although studies suggest that eplerenone crosses the blood brain barrier in rodent models of hypertension and heart failure when given systematically,^10^, ^30^ the dose sufficient to induce sympatho‐inhibitory action in humans has not been established. On a milligram to milligram basis, eplerenone is 50% less potent than spironolactone in lowering BP, though more selective to MR than spironolactone.^31^ We have previously demonstrated that spironolactone mitigates EPR overactivity in SHR to the same degree as eplerenone.^9^ Given this, we did not treat the hypertensive patients participating in this study with spironolactone. Unlike rodents, it is possible that spironolactone may have a greater effect in humans and should be examined in future studies. Second, the potential action of eplerenone on SNA may be evident only when the renin angiotensin system is activated. Our previous studies in hypertensive patients showed that spironolactone alone had no effects on the resting SNA while chlorthalidone, a thiazide diuretic proposed to be a first line diuretic for the treatment of hypertension, induced persistent sympathetic activation in the same hypertensive patients.^32^ However, spironolactone exerts a sympatho‐inhibitory action when given in combination with chlorthalidone in hypertensive patients.^33^ Whether eplerenone exerts a similar influence on SNA at rest or during exercise when given in combination with thiazide diuretics (ie, during renin angiotensin system activation) remains to be evaluated.

Our study is limited by a small sample size. Nevertheless, we demonstrate a clear‐cut increase in SNA during metaboreflex activation during static handgrip exercise and PECA in patients treated with amlodipine. The dose of amlodipine and eplerenone used in our study are relatively small and the results may not be applicable during the use of higher dosages. This is particularly important as prior studies showed that dihydropyridine calcium channel blockers may have mineralocorticoid antagonist properties at higher doses.^34^ This effect is likely not applicable in our study given the low doses of calcium channel blocker used. Renin and aldosterone levels were not measured for this analysis; therefore, the effect of renin and aldosterone levels on the variables in our study is unknown. Exercise intervention was limited to the arm and hand muscles so it is unclear if the results will be reproducible with lower extremity exercise. Nevertheless, our study may have significant clinical implications since amlodipine is proposed as one of the first line drug treatments for hypertension.^17^ Exercise training is an important component of the nonpharmacologic treatment of hypertension.^35^, ^36^ A meta‐analysis of randomized controlled trials showed that BP was reduced by 3.5/2.5 mm Hg with moderate to high intensity endurance training with duration between 100 and 150 min per week.^37^ In the same meta‐analysis, the average BP reduction appears to be larger with isometric resistance training by 10.9/6.2 mm Hg, though the total number of studies were relatively smaller than the endurance training exercise.^38^ Accordingly, a scientific statement from the American Heart Association (AHA) has adopted isometric handgrip and resistance exercise training in addition to endurance exercise training as acceptable modalities of exercise intervention to reduce BP.^35^ The most recent 2017 AHA High Blood Pressure Clinical Practice Guideline has specifically recommended handgrip exercise at 30–40% maximum voluntary contraction, three sessions per week.^17^ Because persistent sympathetic activation is associated with poor prognosis in patients with cardiovascular diseases,^39^, ^40^, ^41^ our study has a potentially significant clinical implication in terms of selecting antihypertensive agents that have the least detrimental effects on the sympathetic neural and hemodynamic responses to isometric exercise.

## 6 CONCLUSIONS

We found that amlodipine, a first line antihypertensive agent, potentiated the rise in SNA and HR during static handgrip and post‐handgrip circulatory arrest. This effect of amlodipine was not observed during dynamic arm cycling exercise, suggesting selective metaboreflex sensitization during static exercise. Contrary to preclinical studies, mineralocorticoid receptor antagonism did not have a significant effect on SNA during static or dynamic exercise in hypertensive humans. Further studies are needed to determine if the effect of amlodipine on metaboreflex represents a class effect and whether higher doses of eplerenone are needed to alter SNA during exercise in humans.

## CONFLICT OF INTEREST

None.

## References

[1] Philipp Th , Distler A , Cordes U . Sympathetic nervous system and blood‐pressure control in essential hypertension. Lancet. 1978;2:959‐963. 81987 10.1016/s0140-6736(78)92526-6

[2] Schütz W , Hörtnagl H , Magometschnigg D . Function of the autonomic nervous system in young. Int J Cardiol. 1986;10:133‐140. 3002992 10.1016/0167-5273(86)90221-4

[3] Smith SA , Williams MA , Leal AK , Mitchell JH , Garry MG . Exercise pressor reflex function is altered in spontaneously hypertensive rats. J Physiol. 2006;577:1009‐1020. 17023501 10.1113/jphysiol.2006.121558 PMC1890389

[4] Mizuno M , Murphy MN , Mitchell JH , Smith SA . Skeletal muscle reflex‐mediated changes in sympathetic nerve activity are abnormal in spontaneously hypertensive rats. Am J Physiol Heart Circulatory Physiol. 2011;300:H968‐77. 10.1152/ajpheart.01145.2010 PMC3064311 21217062

[5] Kaufman MP . Mechanoreceptors and central command. American journal of physiology Heart and circulatory physiology. 2007;292:H117‐8. 16997885 10.1152/ajpheart.01020.2006

[6] Leal AK , Williams MA , Garry MG , Mitchell JH , Smith SA . Evidence for functional alterations in the skeletal muscle mechanoreflex and metaboreflex in hypertensive rats. Am J Physiol Heart Circul Physiol. 2008. 10.1152/ajpheart.01365.2007 PMC2593527 18641268

[7] Vongpatanasin W , Wang Z , Arbique D , et al. Functional sympatholysis is impaired in hypertensive humans. J Physiol. 2011;589:1209‐1220. 21224235 10.1113/jphysiol.2010.203026 PMC3060597

[8] Delaney EP , Greaney JL , Edwards DG , Rose WC , Fadel PJ , Farquhar WB . Exaggerated sympathetic and pressor responses to handgrip exercise in older hypertensive humans: role of the muscle metaboreflex. Am J Physiol Heart Circul Physiol. 2010;299:H1318‐27. 10.1152/ajpheart.00556.2010 PMC2993192 20802135

[9] Greaney JL , Edwards DG , Fadel PJ , Farquhar WB . Rapid onset pressor and sympathetic responses to static handgrip in older hypertensive adults. J Hum Hypertens. 2015;29:402‐408. 25471615 10.1038/jhh.2014.106

[10] Downey RM , Mizuno M , Mitchell JH , Vongpatanasin W , Smith SA . Mineralocorticoid receptor antagonists attenuate exaggerated exercise pressor reflex responses in hypertensive rats. Am J Physiol Heart Circul Physiol. 2017;313:H788‐H794. 10.1152/ajpheart.00155.2017 PMC5668601 28733447

[11] Rahmouni K , Barthelmebs M , Grima M , Imbs J‐L , De Jong W . Brain mineralocorticoid receptor control of blood pressure and kidney function in normotensive rats. Hypertension. 1999;33:1201‐1206. 10334812 10.1161/01.hyp.33.5.1201

[12] Mizuno M , Downey RM , Mitchell JH , Auchus RJ , Smith SA , Vongpatanasin W . Aldosterone and salt loading independently exacerbate the exercise pressor reflex in rats. Hypertension. 2015;66:627‐633. 26195483 10.1161/HYPERTENSIONAHA.115.05810 PMC4537393

[13] Grassi G , Spaziani D , Seravalle G , et al. Effects of amlodipine on sympathetic nerve traffic and baroreflex control of circulation in heart failure. Hypertension. 1999;33:671‐675. 10024325 10.1161/01.hyp.33.2.671

[14] Velasco A , Solow E , Price A , et al. Differential effects of nebivolol vs. metoprolol on microvascular function in hypertensive humans. Am J Physiol Heart Circ Physiol. 2016;311:H118‐24. 27199121 10.1152/ajpheart.00237.2016 PMC4967201

[15] Mark AL , Victor RG , Nerhed C , Wallin BG . Microneurographic studies of the mechanisms of sympathetic nerve responses to static exercise in humans. Circ Res. 1985;57:461‐469. 4028348 10.1161/01.res.57.3.461

[16] White WB , Duprez D , St Hillaire R , et al. Effects of the selective aldosterone blocker eplerenone versus the calcium antagonist amlodipine in systolic hypertension. Hypertension. 2003;41:1021‐1026. 12682082 10.1161/01.HYP.0000067463.13172.EA

[17] Whelton PK , Carey RM , Aronow WS , et al. ACC/AHA/AAPA/ABC/ACPM/AGS/APhA/ASH/ASPC/NMA/PCNA guideline for the prevention, detection, evaluation, and management of high blood pressure in adults: a report of the American college of cardiology/American heart association task force on clinical practice guidelines. Hypertension. 2018;71(6):1269‐1324. 29133354 10.1161/HYP.0000000000000066

[18] Inomata J‐I , Murai H , Kaneko S , et al. Differential effects of azelnidipine and amlodipine on sympathetic nerve activity in patients with primary hypertension. J Hypertens. 2014;32:1898‐1904. 24979307 10.1097/HJH.0000000000000270

[19] Kaufman MP , Hayes SG . The exercise pressor reflex. Clin Auton Res. 2002;12:429‐439. 12598947 10.1007/s10286-002-0059-1

[20] Victor RG , Bertocci LA , Pryor SL , Nunnally RL . Sympathetic nerve discharge is coupled to muscle cell pH during exercise in humans. J Clin Invest. 1988;82:1301‐1305. 3170747 10.1172/JCI113730 PMC442683

[21] Smith SA , Mitchell JH , Garry MG . The mammalian exercise pressor reflex in health and disease. Experim Physiol. 2006;91:89‐102. 10.1113/expphysiol.2005.032367 16282366

[22] Spranger MD , Sala‐Mercado JA , Coutsos M , et al. Role of cardiac output versus peripheral vasoconstriction in mediating muscle metaboreflex pressor responses: dynamic exercise versus postexercise muscle ischemia. Am J Physiol Regul Integr Comp Physiol. 2013;304:R657‐63. 23427084 10.1152/ajpregu.00601.2012 PMC3627949

[23] Bonde‐Petersen F , Rowell LB , Murray RG , et al. Role of cardiac output in the pressor responses to graded muscle ischemia in man. J Appl Physiol Respir Environ Exerc Physiol. 1978;45:574‐580. 711574 10.1152/jappl.1978.45.4.574

[24] Spranger MD , Kaur J , Sala‐Mercado JA , et al. Attenuated muscle metaboreflex‐induced pressor response during postexercise muscle ischemia in renovascular hypertension. Am J Physiol Regul Integr Comp Physiol. 2015;308:R650‐8. 25632024 10.1152/ajpregu.00464.2014 PMC4386005

[25] Becker BF , Möbert J . Low‐dose calcium antagonists reduce energy demand and cellular damage of isolated hearts during both ischemia and reperfusion. Naunyn Schmiedebergs Arch Pharmacol. 1999;360:287‐294. 10543430 10.1007/s002109900053

[26] Gillies HC , Derman EW , Noakes TD . Effects of amlodipine on exercise performance and cardiovascular and skeletal muscle function in physically active hypertensive patients. Clin Drug Invest. 1996;12:135‐145.

[27] Kim D‐S , Yoon C‐H , Lee S‐J , Park S‐Y , Yoo H‐J , Cho H‐J . Changes in voltage‐gated calcium channel alpha(1) gene expression in rat dorsal root ganglia following peripheral nerve injury. Brain Res Mol Brain Res. 2001;96:151‐156. 11731020 10.1016/s0169-328x(01)00285-6

[28] Lee S . Pharmacological inhibition of voltage‐gated Ca(2+) channels for chronic pain relief. Curr Neuropharmacol. 2013;11:606‐620. 24396337 10.2174/1570159X11311060005 PMC3849787

[29] Tang Q , Bangaru MLY , Kostic S , et al. Ca(2)(+)‐dependent regulation of Ca(2)(+) currents in rat primary afferent neurons: role of CaMKII and the effect of injury. J Neurosci. 2012;32:11737‐11749. 22915116 10.1523/JNEUROSCI.0983-12.2012 PMC3723336

[30] Kang Y‐M , Zhang Z‐H , Johnson RF , et al. Novel effect of mineralocorticoid receptor antagonism to reduce proinflammatory cytokines and hypothalamic activation in rats with ischemia‐induced heart failure. Circ Res. 2006;99:758‐766. 16960100 10.1161/01.RES.0000244092.95152.86

[31] Sica DA . The risks and benefits of aldosterone antagonists. Curr Heart Fail Rep. 2005;2:65‐71. 16036053 10.1007/s11897-005-0011-5

[32] Menon DV , Arbique D , Wang Z , Adams‐Huet B , Auchus RJ , Vongpatanasin W . Differential effects of chlorthalidone versus spironolactone on muscle sympathetic nerve activity in hypertensive patients. J Clin Endocrinol Metab. 2009;94:1361‐1366. 19158191 10.1210/jc.2008-2660 PMC2682477

[33] Raheja P , Price A , Wang Z , et al. Spironolactone prevents chlorthalidone‐induced sympathetic activation and insulin resistance in hypertensive patients. Hypertension. 2012;60:319‐325. 22733474 10.1161/HYPERTENSIONAHA.112.194787 PMC3401321

[34] Dietz JD , Du S , Bolten CW , et al. A number of marketed dihydropyridine calcium channel blockers have mineralocorticoid receptor antagonist activity. Hypertension. 2008;51:742‐748. 18250364 10.1161/HYPERTENSIONAHA.107.103580

[35] Brook RD , Appel LJ , Rubenfire M , et al. American Heart Association Professional Education Committee of the Council for High Blood Pressure Research CoC, Stroke Nursing CoE, Prevention and Council on Nutrition PA. Beyond medications and diet: alternative approaches to lowering blood pressure: a scientific statement from the american heart association. Hypertension. 2013;61:1360‐1383. 23608661 10.1161/HYP.0b013e318293645f

[36] Pescatello LS , Franklin BA , Fagard R , et al, American College of Sports M . American college of sports medicine position stand. Exercise and hypertension. Med Sci Sports Exerc. 2004;36:533‐553. 15076798 10.1249/01.mss.0000115224.88514.3a

[37] Cornelissen VA , Smart NA . Exercise training for blood pressure: a systematic review and meta‐analysis. J Am Heart Assoc. 2013;2:e004473. 23525435 10.1161/JAHA.112.004473 PMC3603230

[38] Peri‐Okonny P , Fu Q , Zhang R , et al. Exercise, the brain, and hypertension. Curr Hypertens Rep. 2015;17:82. 26341655 10.1007/s11906-015-0593-6

[39] Masuo K . Sympathetic nerve hyperactivity precedes hyperinsulinemia and blood pressure elevation in a young, nonobese Japanese population. Am J Hypertens. 1997;10:77‐83. 9008251 10.1016/s0895-7061(96)00303-2

[40] Masuo K , Kawaguchi H , Mikami H , Ogihara T , Tuck ML . Serum uric acid and plasma norepinephrine concentrations predict subsequent weight gain and blood pressure elevation. Hypertension. 2003;42:474‐480. 12953019 10.1161/01.HYP.0000091371.53502.D3

[41] Cohn JN , Levine TB , Olivari MT , et al. Plasma norepinephrine as a guide to prognosis in patients with chronic congestive heart failure. N Engl J Med. 1984;311:819‐823. 6382011 10.1056/NEJM198409273111303

